# Hemorrhagic Shock Revealing Rupture of Splenic Artery Pseudoaneurysm Three Years After Post-Traumatic Pancreatitis

**DOI:** 10.7759/cureus.15678

**Published:** 2021-06-16

**Authors:** Karim El Aidaoui, Ahmed Bensaad, Jihane Habi, Khalid El Yamani, Chafik El Kettani

**Affiliations:** 1 Anesthesia and Critical Care, Cheikh Khalifa International University Hospital, Mohammed VI University of Health Sciences, Casablanca, MAR; 2 Surgical Gastroenterology, Cheikh Khalifa International University Hospital, Mohammed VI University of Health Sciences, Casablanca, MAR; 3 Radiology, Cheikh Khalifa International University Hospital, Mohamed VI University of Health Sciences, Casablanca, MAR

**Keywords:** pseudoaneurysm of splenic artery, traumatic pancreatitis, hemorrhagic shock, splenic artery aneurysm rupture, pancreatic pseudocyst, splenic artery

## Abstract

Splenic artery pseudoaneurysm (SAP) is an uncommon entity but extremely serious, given the high mortality rate if untreated. Only a limited literature reports association with post-traumatic pancreatitis. We report the case of a 30-year-old man, who was brought to the emergency department (ED) for acute confusion. His past medical history includes trauma of right hypochondriac and epigastric regions, three years ago. Three days before his admission to the hospital, he experienced abdominal pain with nausea and vomiting, without transit disorders or fever. When examined, the patient was disoriented, pale with profuse sweating, cold extremities, and a temperature of 36.3°C. Blood pressure was 75/51 mmHg, heart rate was 126 beats per minute, and oxygen saturation was 96% on room air. The abdominal exam detected generalized abdominal sensitivity. A CT angiography of the abdomen revealed hemoperitoneum of medium abundance, with extravasation of the contrast product from the splenic artery. The size of the spleen was normal with a lower polar hypodense area. In addition, a pancreas of normal size, steady outlinings, seat of bilobed cystic formation suggested a pancreatic pseudocyst. This led us to suspect a rupture of a pseudoaneurysm of the splenic artery. A laparotomy was performed and showed an estimated 2 L hemoperitoneum. Active bleeding was noted from an SAP in the mid-portion of the splenic artery, next to the pancreatic pseudocyst. Ligation of the splenic artery and splenectomy was carried out. The patient was discharged home on the 10th post-operative day. Our case highlights an uncommon cause of hemorrhagic shock, but critical to recognize. Indeed, ruptured SAP needs to be promptly detected and managed, to avoid fatal complications if left untreated.

## Introduction

Splenic artery pseudoaneurysms (SAPs) are uncommon in healthy young patients but critical to recognize. These SAPs are often incidentally detected due to the widespread use of high-resolution imaging techniques [1]. Hemorrhage and abdominal pain are the most common manifestations [2]. SAP can be life-threatening if ruptured. Only limited literature describes association with post-traumatic pancreatitis. We report the case of a spontaneous SAP rupture in a young man with a past history of abdominal trauma, revealed by hemorrhagic shock and treated successfully with traditional open surgery.

## Case presentation

Mr M.Z. is a 30-year-old man, who was brought to the emergency department (ED) for acute confusion. His past medical history includes trauma of right hypochondriac and epigastric regions, three years ago. Three days before his admission to the hospital, he experienced abdominal pain with nausea and vomiting, without transit disorders or fever. When examined, the patient was disoriented, pale with profuse sweating, cold extremities, and a temperature of 36.3°C. His vital signs were unstable: blood pressure was 75/51 mmHg, heart rate was 126 beats per minute, and oxygen saturation was 96% on room air. The abdominal exam detected generalized abdominal sensitivity with free hernial orifice and digital rectal examination without special features.

**Figure 1:**
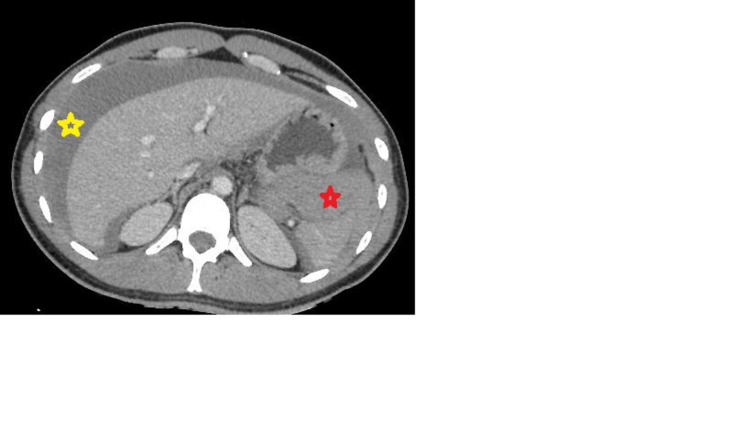
An axial view of a contrast-enhanced portal venous phase CT scan, showing a perihepatic fluid effusion (yellow star) and a perisplenic hemoperitoneum (red star).

The patient was resuscitated with 2 L of normal saline and was given norepinephrine to stabilize his hemodynamic condition before radiological explorations. Cerebral MRI was performed but did not reveal any abnormality. A CT angiography of the abdomen revealed hemoperitoneum of medium abundance (Figure 1), with extravasation of the contrast product from the splenic artery.

**Figure 2:**
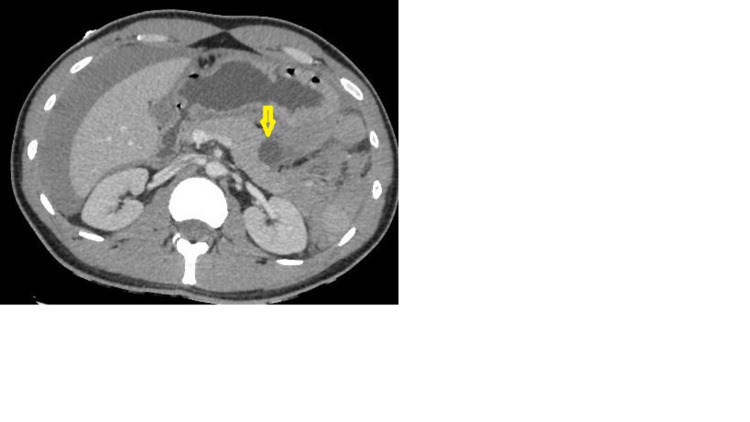
An axial view of a contrast-enhanced portal venous phase CT scan, showing in the corporeo-caudal junction of the pancreas, a rounded formation well limited and not taking the contrast, suggesting a pancreatic pseudocyst (yellow arrow).

The size of the spleen was normal with a lower polar hypodense area. The pancreas had a normal size, steady outlinings, and included a bilobed cystic formation measuring 19 mm x 24 mm suggesting a pancreatic pseudocyst (Figure 2).

**Figure 3:**
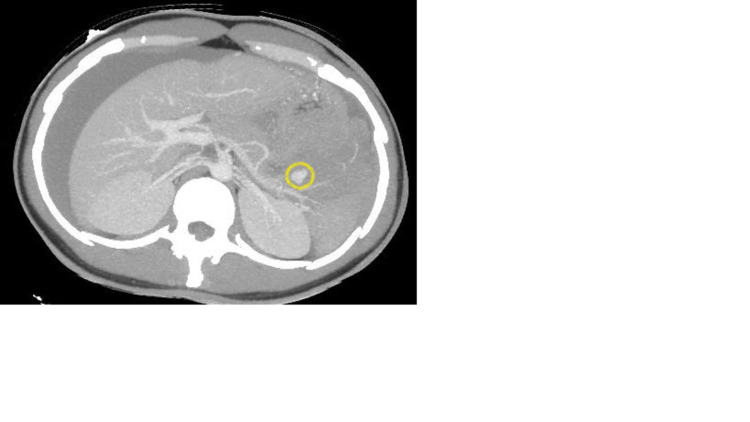
An axial view of a contrast-enhanced portal venous phase CT scan and MIP reconstruction showing a pseudoaneurysm (yellow circle) of a branch of the splenic artery without sign of active bleeding. MIP, maximum intensity projection

**Figure 4:**
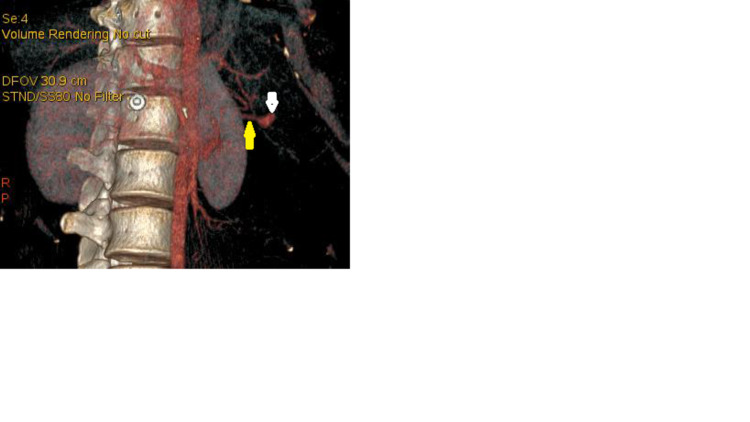
Vascular reconstruction showing pseudoaneurysm (white arrow) communicating with a branch of the splenic artery (yellow arrow).

This led us to suspect a rupture of a pseudoaneurysm of the splenic artery (Figures 3-4) complicated by splenic infarction. 

Due to active bleeding and the shock state, surgical exploration was advised. The pre-operative assessment showed: hemoglobin at 6.2 g/dL, platelets at 160 x 109/L, prothrombin time (PT) at 65%, activated partial thromboplastin time (aPTT) at 35 s, and normal fibrinogen. After stabilization of vital signs with fluid infusion and norepinephrine, the patient was taken for an emergency laparotomy. When exploring, a hemoperitoneum of 2 L was detected. Active bleeding was noted from an SAP in the mid-portion of the splenic artery, next to the pancreatic cyst. The fibrosis caused by this cyst made the dissection of the ruptured splenic artery branch difficult and time-consuming. The surgical team, therefore, performed a splenic artery ligation and a splenectomy. A tissue sample was taken from the wall of the pancreatic cyst to confirm its nature. The patient was transfused with four units of packed red blood cells (RBCs) and four units of fresh frozen plasma (FFP) during the surgery.

The patient’s recovery was uneventful with post-operative hemoglobin of 8.5 g/dL. Biopsy taken from the wall of the pancreatic cyst showed no epithelium and confirmed that it was in fact a pseudocyst. Given the small size of the pseudocyst, and since there was no pancreatic fistula in the post-operative course, we opted for conservative management with periodic radiological monitoring. The patient was discharged home on the 10th post-operative day, after receiving post-splenectomy vaccinations. No complications were seen at three months following discharge from the hospital.

## Discussion

Peripancreatic arteries pseudoaneurysms are rare entities. Pancreatitis is the most common etiology, followed by abdominal trauma and iatrogenic causes [3]. SAP is the most frequent because of the proximity of the splenic artery to the pancreas. But SAPs are far less common than true aneurysms of the splenic artery [4]. Other arteries that may be affected are the: gastroduodenal, pancreaticoduodenal, hepatic, and left gastric arteries [5]. In true aneurysms, the wall is composed of intima, media, and adventitia. In comparison, pseudoaneurysms have a thin wall [6] containing only intima and media, which explains their higher risk of spontaneous rupture and their higher mortality rate [7]. There is no correlation between the size of a pseudoaneurysm and rupture in the literature [3]. The mechanisms that lead to pseudoaneurysm formation include peripancreatic inflammation and enzymatic autodigestion of the arterial wall or a pancreatic pseudocyst eroding into a visceral artery, thus converting the pseudocyst into a large pseudoaneurysm [8].

The mechanism for the SAP in the case of our patient is thought to be secondary to the abdominal trauma that occurred three years ago. Biopsy taken from the wall of the cyst showed no epithelium and confirmed that it was a pseudocyst. We formulated the retrospective hypothesis that abdominal trauma caused undetected pancreatitis and subsequently the pseudocyst formation. As the lesion was adjacent to the branch of the splenic artery, we think that chronic erosion led to SAP formation.

The diagnosis of SAP is difficult in the presence of pancreatic pseudocyst because the CT scan of the abdomen may miss small pseudoaneurysms. Abdominal CT angiography is more efficient to establish a definitive diagnosis when the suspicion of SAP is high [9]. The combination of pancreatic pseudocysts and pseudoaneurysms is difficult to manage and is responsible for high morbidity and mortality [7]. Indeed, pancreatic pseudocysts may cause major vessel erosion with pseudoaneurysm formation which may result in severe bleeding into the peritoneal cavity or the retroperitoneum [10]. The mortality rate of untreated patients with bleeding pseudoaneurysms is between 90% and 100% and even with aggressive treatment, the mortality is between 18% and 29% [7].

The traditional approach to the treatment of pancreatic pseudoaneurysms has been surgery. Traditional open surgery either splenectomy with or without distal pancreatectomy, or splenic artery ligation, and SAP resection were the main forms of treatment. Over the past three decades, with improvement in interventional radiology, endovascular therapy has become the accepted and preferred treatment modality in hemodynamically stable patients [4]. Indeed, a number of reports describe the successful resolution of bleeding pseudoaneurysm with angioembolization [8], which is recommended as the initial therapy for hemodynamically stable patients. In our case, since the patient was never really stable due to massive bleeding, the surgical team opted for traditional open surgery with splenic artery ligation and splenectomy. However, surgery should be reserved for hemodynamically unstable patients with active bleeding; for failed embolization; and for other secondary complications such as infection or extrinsic compression [7].

## Conclusions

Splenic artery pseudoaneurysms are at higher risk for spontaneous rupture and significant morbidity in comparison with true aneurysms. Our patient presented a hemorrhagic shock due to the rupture of SAP, three years after an unexplored abdominal trauma complicated by a pancreatic pseudocyst. The combination of SAP and pancreatic pseudocysts is rare and difficult to manage. The SAP rupture needs to be promptly detected and managed, given its high mortality rate if untreated. Minimal invasive procedures like angioembolization are to be attempted before the major invasive surgical procedure once the patient is hemodynamically stabilized. However, the surgery has its own role in unstable patients with active bleeding.
